# Supplementary material for: Study of the Association between VEGF Polymorphisms and the Risk of Coronary Artery Disease in Koreans
Source: J Pers Med. 2022 May 7;12(5):761. doi: 10.3390/jpm12050761 (PMC9144104; doi:10.3390/jpm12050761)
Supplement: Supplementary file 1 [file jpm-12-00761-s001.zip › jpm-1691005-supplementary.pdf]

Table S1. Information of *VEGF* polymorphisms primer set for PCP-RFLP, real-time PCR

**Method**	**Genotype**	**Reference SNP ID**	**Primer sequence**	**Annealing condition**	**Restriction enzyme**	**RFLP condition**
PCR-RFLP	*VEGF* +936C>T	rs1570360	Forward: 5'- CGC GTG TCT CTG GAC AGA GTT TCC -3'<br>Reverse: 5'- CGG GGA CAG GCG AGC TTC AG -3'	59℃, with 35 cycles	*Mnl* Ⅰ	incubation for 16hr at 37℃
	VEGF +936C>T	rs3025039	Forward: 5'– AAG GAA GAG GAG ACT CTG CGC AGA GC –3'<br>Reverse: 5'– TAA ATG TAT GTA TGT GGG TGG GTG TGT CTA CAG G –3'	68℃, with 35 cycles	*Nla*Ⅲ	incubation for 16hr at 37℃
Taqman real-time PCR	*VEGF* +1451C>T	rs3025040	Forward: 5'– ACG GAC AGA AAG ACA GAT CAC AG –3'<br>Reverse: 5'– CCC AAA GCA CAG CAA TGT C –3'<br>C probe: 5'- [FAM]-TGA GGA CAC C GGC TCT GAC C-[TAMRA] -3'<br>T probe: 5'- [JOE]-TGA GGA CAC T GGC TCT GAC C-[TAMRA] -3'	57℃, with 50 cycles		
	VEGF +1612G>A	rs10434	Forward:5'– TTC GCT TAC TCT CAC CTG CTT C –3'<br>Reverse:5'– GCT GTC ATG GGC TGC TTC T –3'<br>G probe: 5'- [FAM]-CCC AGG AG G CCA CTG GCA-[TAMRA] -3'<br>A probe: 5'- [[JOE]-CCC AGG AG A CCA CTG GCA-[TAMRA] -3'	65℃,with 50 Cycles		
	VEGF +1725G>A	rs3025053	Forward: 5'– CAT GAC AGC TCC CCT TCC T –3'<br>Reverse: 5'– TGG TTT CAA TGG TGT GAG GAC –3'<br>G probe: 5'- [FAM]-CTT CCT GGG G TGC AGC CTA A-[TAMRA] -3'<br>A probe: 5'- [JOE]-CTT CCT GGG A TGC AGC CTA A-[TAMRA] -3'	68℃, with 35 cycles		

PCR, polymerase chain reaction, RFLP, restriction fragment length polymorphism

Table S2. Comparison of allele frequency in recruited samples and variable population of 1000 genomes.

SNP	Allele	Control	CAD	EAS	CDX	CHB	CHS	JPT	KHV
rs1570360	A	0.838	0.855	0.184	0.167	0.175	0.176	0.168	0.232
	G	0.162	0.145	0.816	0.833	0.825	0.824	0.832	0.768
rs833061	T	0.758	0.766	0.717	0.763	0.728	0.733	0.663	0.702
	C	0.242	0.234	0.283	0.237	0.272	0.267	0.337	0.298
rs3025039	C	0.803	0.820	0.830	0.866	0.816	0.790	0.841	0.843
	T	0.197	0.180	0.827	0.849	0.816	0.790	0.841	0.843
rs3025040	C	0.812	0.824	0.170	0.134	0.184	0.210	0.159	0.157
	T	0.188	0.176	0.173	0.151	0.184	0.210	0.159	0.157
rs10434	G	0.841	0.826	0.780	0.672	0.791	0.800	0.865	0.758
	A	0.159	0.174	0.220	0.328	0.209	0.200	0.135	0.242
rs3025053	G	0.947	0.932	0.864	0.758	0.913	0.862	0.962	0.813
	A	0.053	0.068	0.136	0.242	0.087	0.138	0.038	0.187

EAS, east Asian; CDX, Chinese Dai in Xishuangbanna, China; CHB, Han Chinese in Beijing, China; CHS, Southern Han Chinese; JPT, Japanese in Tokyo, Japan; KHV, Kinh in Ho Chi Minh City, Vietnam

Table S3. Comparison of genotype frequencies of *VEGF* polymorphisms according to gender.

Genotype	Male control subjects (n=172)	Male CAD patients (n=201)	AOR (95% CI)	*P*	FDR-*P*	Female control subjects (n=250)	Female CAD patients (n=262)	AOR (95% CI)	*P*	FDR-*P*
VEGF -1154G>A										
GG	118 (68.6)	145 (72.1)	1.000 (reference)			175 (70.0)	195 (74.4)	1.000 (reference)		
GA	49 (28.5)	50 (24.9)	0.895 (0.555-1.442)	0.648	0.972	72 (28.8)	62 (23.7)	0.787 (0.517-1.195)	0.261	0.392
AA	5 (2.9)	6 (3.0)	0.744 (0.202-2.736)	0.656	0.811	3 (1.2)	5 (1.9)	1.563 (0.336-7.270)	0.569	0.711
Dominant (GG vs. GA + AA)			0.883 (0.557-1.400)	0.597	0.896			0.815 (0.541-1.229)	0.330	0.480
Recessive (GG + GA vs. AA)			0.759 (0.206-2.796)	0.678	0.876			1.637 (0.356-7.516)	0.526	0.658
HWE-*P*	0.974	0.51				0.138	0.978			
VEGF -1498T>C										
TT	87 (50.6)	117 (58.2)	1.000 (reference)			154 (61.6)	150 (57.3)	1.000 (reference)		
TC	76 (44.2)	75 (37.3)	0.716 (0.463-1.100)	0.133	0.549	82 (32.8)	100 (38.2)	1.172 (0.792-1.735)	0.427	0.512
CC	9 (5.2)	9 (4.5)	0.547 (0.190-1.578)	0.265	0.663	14 (5.6)	12 (4.6)	0.966 (0.410-2.278)	0.937	0.937
Dominant (TT vs. TC + CC)			0.702 (0.460-1.071)	0.100	0.546			1.145 (0.786-1.669)	0.480	0.480
Recessive (TT + TC vs. CC)			0.662 (0.240-1.825)	0.425	0.876			0.918 (0.393-2.143)	0.843	0.843
HWE-*P*	0.140	0.486				0.484	0.361			
VEGF +936C>T										
CC	119 (69.2)	129 (64.2)	1.000 (reference)			154 (61.6)	182 (69.5)	1.000 (reference)		
CT	51 (29.7)	65 (32.3)	1.145 (0.725-1.808)	0.561	0.972	81 (32.4)	72 (27.5)	0.638 (0.423-0.963)	0.032	0.159
TT	2 (1.2)	7 (3.5)	3.263 (0.611-17.426)	0.167	0.663	15 (6.0)	8 (3.1)	0.374 (0.141-0.987)	0.047	0.108
Dominant (CC vs. CT + TT)			1.224 (0.784-1.910)	0.375	0.750			0.593 (0.399-0.881)	0.010	0.054
Recessive (CC + CT vs. TT)			3.240 (0.626-16.781)	0.161	0.805			0.431 (0.166-1.122)	0.085	0.172
HWE-*P*	0.174	0.733				0.327	0.787			
VEGF +1451C>T										
CC	123 (71.5)	131 (65.2)	1.000 (reference)			160 (64.0)	184 (70.2)	1.000 (reference)		
CT	43 (25.0)	63 (31.3)	1.378 (0.859-2.209)	0.183	0.549	76 (30.4)	70 (26.7)	0.662 (0.436-1.005)	0.053	0.159
TT	6 (3.5)	7 (3.5)	1.158 (0.348-3.854)	0.811	0.811	14 (5.6)	8 (3.1)	0.396 (0.148-1.060)	0.065	0.108
Dominant (CC vs. CT + TT)			1.362 (0.866-2.142)	0.182	0.546			0.617 (0.413-0.922)	0.018	0.054
Recessive (CC + CT vs. TT)			1.098 (0.338-3.567)	0.876	0.876			0.448 (0.171-1.177)	0.103	0.172
HWE-*P*	0.363	0.865				0.222	0.671			
VEGF +1612G>A										
GG	119 (69.2)	140 (69.7)	1.000 (reference)			179 (71.6)	180 (68.7)	1.000 (reference)		
GA	48 (27.9)	54 (26.9)	0.973 (0.606-1.563)	0.911	0.972	66 (26.4)	71 (27.1)	1.050 (0.688-1.603)	0.822	0.822
AA	5 (2.9)	7 (3.5)	1.317 (0.393-4.412)	0.655	0.811	5 (2.0)	11 (4.2)	3.020 (0.977-9.339)	0.055	0.108
Dominant (GG vs. GA + AA)			0.984 (0.624-1.552)	0.946	0.972			1.171 (0.780-1.759)	0.447	0.48
Recessive (GG + GA vs. AA)			1.109 (0.334-3.688)	0.866	0.876			2.828 (0.927-8.630)	0.068	0.172
HWE-*P*	0.952	0.531				0.703	0.245			
VEGF +1725G>A										
GG	150 (87.2)	175 (87.1)	1.000 (reference)			227 (90.8)	228 (87.0)	1.000 (reference)		
GA	22 (12.8)	26 (12.9)	1.011 (0.537-1.904)	0.972	0.972	23 (9.2)	31 (11.8)	1.391 (0.765-2.529)	0.2	0.392
AA	0 (0.0)	0 (0.0)	N/A	N/A	N/A	0 (0.0)	3 (1.1)	N/A	N/A	N/A
Dominant (GG vs. GA + AA)			1.011 (0.537-1.904)	0.972	0.972			1.532 (0.850-2.760)	0.156	0.312
Recessive (GG + GA vs. AA)			N/A	N/A	N/A			N/A	N/A	N/A
HWE-*P*	0.370	0.327				0.446	0.111			

CAD, coronary artery disease; COR, crude odds ratio; CI, confidence interval; AOR, adjusted odds ratio. AOR: Adjusted by age, gender, hypertension, diabetes mellitus, hyperlipidemia, and smoking status.

Table S4. Haplotype analysis of *VEGF* polymorphisms in CAD patients and control subjects.

Haplotype	Control subjects (2n=844)	CAD patients (2n=926)	OR (95% CI)	*P*
VEGF -1154G>A/-1498T>C/+936C>T				
G-T-C	509 (60.3)	607 (65.6)	1.000 (reference)	
**A**-T-**C**	24 (2.8)	2 (0.2)	0.070 (0.016-0.297)	<0.0001
A-T-T	6 (0.7)	0 (0.0)	0.065 (0.004-1.149)	0.009
VEGF -1154G>A/-1498T>C/+1451C>T				
G-T-C	512 (60.7)	608 (65.6)	1.000 (reference)	
A-T-C	27 (3.2)	2 (0.2)	0.062 (0.015-0.264)	<0.0001
VEGF -1154G>A/-1498T>C/+1612G>A				
G-T-G	500 (59.2)	571 (61.7)	1.000 (reference)	
A-T-G	29 (3.4)	1 (0.2)	0.030 (0.004-0.223)	<0.0001
VEGF -1154G>A/-1498T>C/+1725G>A				
G-T-G	577 (68.3)	652 (70.4)	1.000 (reference)	
**A**-T-G	30 (3.5)	1 (0.1)	0.030 (0.004-0.217)	<0.0001
VEGF -1154G>A/+936C>T/+1451C>T				
G-C-C	572 (67.8)	657 (70.9)	1.000 (reference)	
A-T-C	6 (0.7)	0 (0.0)	0.067 (0.004-1.192)	0.010
VEGF -1498T>C/+936C>T/+1451C>T				
T-C-C	526 (62.4)	607 (65.6)	1.000 (reference)	
T-T-C	13 (1.5)	3 (0.4)	0.200 (0.057-0.706)	0.006
VEGF -1498T>C/+936C>T/+1725G>A				
T-C-G	504 (59.7)	551 (59.5)	1.000 (reference)	
**T**-C-**A**	29 (3.4)	58 (6.3)	1.829 (1.153-2.904)	0.010
VEGF -1498T>C/+1451C>T/+1725G>A				
T-C-G	507 (60.1)	551 (59.5)	1.000 (reference)	
**T**-C-**A**	32 (3.8)	58 (6.3)	1.668 (1.065-2.611)	0.024
VEGF -1498T>C/+1612G>A/+1725G>A				
T-G-G	531 (62.9)	572 (61.8)	1.000 (reference)	
**T-A-A**	33 (4.0)	59 (6.3)	1.660 (1.067-2.583)	0.024
VEGF +936C>T/+1451C>T/+1612G>A				
C-C-G	536 (63.5)	599 (64.7)	1.000 (reference)	
C-T-G	9 (1.1)	1 (0.2)	0.099 (0.013-0.788)	0.009
T-C-G	15 (1.8)	4 (0.4)	0.239 (0.079-0.724)	0.006
VEGF +936C>T/+1451C>T/+1725G>A				
C-C-G	626 (74.2)	695 (75.0)	1.000 (reference)	
C-T-G	9 (1.0)	1 (0.1)	0.100 (0.013-0.793)	0.009
T-C-G	15 (1.8)	6 (0.7)	0.360 (0.139-0.935)	0.029
VEGF -1154G>A/-1498T>C				
G-T	610 (72.3)	707 (76.3)	1.000 (reference)	
A-T	30 (3.5)	2 (0.2)	0.058 (0.014-0.242)	<0.0001
VEGF -1498T>C/+1725G>A				
T-G	607 (71.9)	650 (70.2)	1.000 (reference)	
T-A	33 (3.9)	59 (6.3)	1.670 (1.075-2.593)	0.021
VEGF +936C>T/+1451C>T				
C-C	669 (79.3)	757 (81.8)	1.000 (reference)	
C-T	9 (1.1)	2 (0.2)	0.196 (0.042-0.913)	0.021
T-C	16 (1.9)	6 (0.7)	0.331 (0.129-0.852)	0.016

Note: CAD, coronary artery disease; OR, odd ratio; CI, confidence interval.

Table S5. Genotype combination of VEGF polymorphisms in CAD patients and control subjects.

Genotype combination	Control subjects (n=422)	CAD patients (n=463)	AOR (95% CI)	*P*
GG/TT/CC	154 (36.5)	192 (45.5)		
GG/TT/CT	64 (15.2)	70 (16.6)	0.828 (0.545-1.260)	0.378
GG/TT/TT	5 (1.2)	2 (0.5)	0.322 (0.054-1.934)	0.215
GG/TC/CC	36 (8.5)	39 (9.2)	0.827 (0.484-1.413)	0.486
GG/TC/CT	22 (5.2)	28 (6.6)	1.134 (0.594-2.166)	0.704
GG/TC/TT	2 (0.5)	7 (1.7)	3.757 (0.736-19.169)	0.111
GG/CC/CC	5 (1.2)	1 (0.2)	0.161 (0.016-1.591)	0.118
GG/CC/CT	5 (1.2)	0 (0.0)	N/A	N/A
GG/CC/TT	1 (0.2)	0 (0.0)	N/A	N/A
GA/TT/CC	12 (2.8)	3 (0.7)	0.224 (0.059-0.845)	0.027
GA/TT/CT	5 (1.2)	0 (0.0)	N/A	N/A
GA/TC/CC	57 (13.5)	68 (16.1)	0.907 (0.590-1.395)	0.658
GA/TC/CT	25 (5.9)	32 (7.6)	0.843 (0.461-1.541)	0.579
GA/TC/TT	7 (1.7)	4 (0.9)	0.230 (0.054-0.988)	0.048
GA/CC/CC	6 (1.4)	4 (0.9)	0.446 (0.113-1.760)	0.249
GA/CC/CT	5 (1.2)	4 (0.9)	0.533 (0.132-2.158)	0.378
GA/CC/TT	1 (0.2)	0 (0.0)	N/A	N/A
AA/TT/CT	1 (0.2)	0 (0.0)	N/A	N/A
AA/TC/CC	2 (0.5)	0 (0.0)	N/A	N/A
AA/TC/CT	4 (0.9)	0 (0.0)	N/A	N/A
AA/CC/CC	1 (0.2)	4 (0.9)	2.639 (0.261-26.674)	0.411
AA/CC/CT	1 (0.2)	3 (0.7)	2.989 (0.292-30.571)	0.356
AA/CC/TT	1 (0.2)	2 (0.5)	2.075 (0.165-26.177)	0.572

Note: CAD, coronary artery disease; AOR, adjusted odd ratio; CI, confidence interval.

AOR; adjusted by age, gender, hypertension, diabetes mellitus, hyperlipidemia, and smoking

Table S6. Genotype combination of *VEGF* polymorphisms in CAD patients and controls subjects.

Genotype combination	Control subjects (n=422)	CAD patients (n=463)	AOR (95% CI)	*P*
VEGF -1154G>A/-1498T>C				
GG/TT	222 (52.6)	265 (57.2)	1.000 (reference)	
GG/TC	61 (14.5)	73 (15.8)	0.839 (0.558-1.261)	0.399
GG/CC	10 (2.4)	2 (0.4)	0.143 (0.030-0.689)	0.015
GA/TT	18 (4.3)	2 (0.4)	0.067 (0.014-0.315)	0.001
GA/TC	91 (21.6)	102 (22.0)	0.930 (0.656-1.318)	0.682
GA/CC	12 (2.8)	8 (1.7)	0.645 (0.245-1.697)	0.374
AA/TT	1 (0.2)	0 (0.0)	N/A	0.998
AA/TC	6 (1.4)	0 (0.0)	N/A	0.997
AA/CC	1 (0.2)	11 (2.4)	7.753 (0.944-63.705)	0.057
VEGF -1154G>A/+1612G>A				
GG/GG	199 (47.2)	237 (51.2)	1.000 (reference)	
GG/GA	86 (20.4)	87 (18.8)	0.795 (0.550-1.149)	0.222
GG/AA	8 (1.9)	16 (3.5)	1.826 (0.749-4.452)	0.185
GA/GG	91 (21.6)	74 (16.0)	0.673 (0.463-0.978)	0.038
GA/GA	28 (6.6)	36 (7.8)	1.096 (0.637-1.886)	0.740
GA/AA	2 (0.5)	2 (0.4)	1.034 (0.137-7.818)	0.974
AA/GG	8 (1.9)	9 (1.9)	0.776 (0.275-2.185)	0.630
AA/GA	0 (0.0)	2 (0.4)	N/A	0.998
AA/AA	0 (0.0)	0 (0.0)	N/A	N/A
VEGF +936C>T/+1451C>T				
CC/CC	268 (63.5)	309 (66.7)	1.000 (reference)	
CC/CT	4 (0.9)	2 (0.4)	0.326 (0.052-2.068)	0.234
CC/TT	1 (0.2)	0 (0.0)	N/A	0.998
CT/CC	14 (3.3)	6 (1.3)	0.342 (0.125-0.940)	0.038
CT/CT	115 (27.3)	131 (28.3)	0.913 (0.668-1.247)	0.567
CT/TT	3 (0.7)	0 (0.0)	N/A	0.998
TT/CC	1 (0.2)	0 (0.0)	N/A	0.998
TT/CT	0 (0.0)	0 (0.0)	N/A	N/A
TT/TT	16 (3.8)	15 (3.2)	0.730 (0.336-1.585)	0.426
VEGF +1612G>A/+1725G>A				
GG/GG	298 (70.6)	320 (69.1)	1.000 (reference)	
GG/GA	0 (0.0)	0 (0.0)	N/A	N/A
GG/AA	0 (0.0)	0 (0.0)	N/A	N/A
GA/GG	70 (16.6)	79 (17.1)	1.008 (0.695-1.463)	0.967
GA/GA	44 (10.4)	46 (9.9)	0.946 (0.597-1.497)	0.812
GA/AA	0 (0.0)	0 (0.0)	N/A	N/A
AA/GG	9 (2.1)	4 (0.9)	0.444 (0.131-1.507)	0.193
AA/GA	1 (0.2)	11 (2.4)	12.061 (1.523-95.491)	0.018
AA/AA	0 (0.0)	3 (0.6)	N/A	N/A

Note: CAD, coronary artery disease; AOR, adjusted odd ratio; CI, confidence interval.

AOR; adjusted by age, gender, hypertension, diabetes mellitus, hyperlipidemia, and smoking.

Table S7. Combinatorial effects of *VEGF* genotypes with individual clinical factors for CAD.

Variables		*VEGF* -1154GG		*VEGF* -1154 GA+AA		*VEGF* -1498 TT		*VEGF* -1498 TC+CC		*VEGF* +936 CC		*VEGF* +936 CT+TT	
		AOR (95% CI)	*P*	AOR (95% CI)	*P*	AOR (95% CI)	*P*	AOR (95% CI)	*P*	AOR (95% CI)	*P*	AOR (95% CI)	*P*
HTN													
	No	1.000 (Reference)		0.783 (0.513-1.193)	0.255	1.000 (Reference)		0.924 (0.629-1.356)	0.685	1.000 (Reference)		0.851 (0.565-1.281)	0.439
	Yes	1.841 (1.309-2.589)	0.0004	1.645 (1.055-2.564)	0.028	2.067 (1.409-3.032)	0.0002	1.731 (1.154-2.597)	0.008	2.011 (1.417-2.853)	0.0001	1.539 (1.012-2.340)	0.044
DM													
	No	1.000 (Reference)		0.809 (0.579-1.131)	0.215	1.000 (Reference)		0.939 (0.689-1.281)	0.692	1.000 (Reference)		0.864 (0.624-1.195)	0.377
	Yes	2.311 (1.513-3.531)	0.0001	2.214 (1.127-4.350)	0.021	2.620 (1.590-4.317)	0.0002	2.113 (1.257-3.553)	0.005	2.553 (1.600-4.075)	0.0001	1.841 (1.063-3.188)	0.030
Hemoglobin A1c (%)													
	<6.5	1.000 (Reference)		0.800 (0.573-1.118)	0.192	1.000 (Reference)		0.964 (0.709-1.310)	0.813	1.000 (Reference)		0.839 (0.609-1.157)	0.285
	≥6.5	4.388 (2.443-7.881)	<0.0001	4.738 (2.094-10.722)	0.0002	5.388 (2.715-10.692)	<0.0001	4.514 (2.208-9.230)	<0.0001	4.677 (2.502-8.742)	<0.0001	3.051 (1.463-6.361)	0.003
Smoking status													
	No	1.000 (Reference)		0.781 (0.538-1.134)	0.194	1.000 (Reference)		1.018 (0.722-1.436)	0.920	1.000 (Reference)		0.745 (0.519-1.070)	0.111
	Yes	0.844 (0.557-1.278)	0.423	0.878 (0.510-1.513)	0.640	0.941 (0.592-1.495)	0.796	0.752 (0.460-1.230)	0.256	0.985 (0.646-1.503)	0.946	0.989 (0.590-1.660)	0.967
BMI													
	<25	1.000 (Reference)		0.917 (0.628-1.341)	0.656	1.000 (Reference)		1.157 (0.818-1.637)	0.411	1.000 (Reference)		1.172 (0.817-1.680)	0.388
	≥25	3.022 (2.117-4.314)	<0.0001	2.092 (1.270-3.448)	0.004	3.486 (2.335-5.204)	<0.0001	2.255 (1.437-3.541)	0.0004	3.586 (2.454-5.242)	<0.0001	1.974 (1.232-3.163)	0.005
Hyperlipidemia													
	No	1.000 (Reference)		0.730 (0.512-1.041)	0.082	1.000 (Reference)		0.780 (0.564-1.079)	0.134	1.000 (Reference)		0.743 (0.529-1.042)	0.085
	Yes	1.153 (0.792-1.679)	0.457	1.455 (0.838-2.528)	0.183	1.052 (0.691-1.600)	0.815	1.548 (0.950-2.523)	0.080	1.136 (0.769-1.678)	0.523	1.308 (0.774-2.210)	0.315
LDL-cholesterol (mg/dL)													
	<130	1.000 (Reference)		0.743 (0.521-1.060)	0.101	1.000 (Reference)		0.868 (0.626-1.204)	0.397	1.000 (Reference)		0.799 (0.569-1.124)	0.197
	≥130	3.217 (1.918-5.394)	<0.0001	10.204 (4.205-24.764)	<0.0001	5.257 (2.718-10.168)	<0.0001	4.049 (2.130-7.697)	<0.0001	4.238 (2.405-7.469)	<0.0001	5.427 (2.578-11.424)	<0.0001
MetS													
	No	1.000 (Reference)		1.034 (0.670-1.596)	0.880	1.000 (Reference)		1.041 (0.695-1.559)	0.846	1.000 (Reference)		1.064 (0.698-1.620)	0.774
	Yes	2.764 (1.932-3.954)	<0.0001	1.816 (1.125-2.933)	0.015	2.674 (1.796-3.981)	<0.0001	2.290 (1.462-3.585)	0.0003	2.988 (2.050-4.356)	<0.0001	1.973 (1.240-3.141)	0.004
Folate (nmol/L)													
	≥4.0	1.000 (Reference)		0.854 (0.615-1.185)	0.344	1.000 (Reference)		0.945 (0.699-1.279)	0.715	1.000 (Reference)		0.868 (0.633-1.189)	0.377
	<4.0	2.158 (1.322-3.522)	0.002	1.861 (0.867-3.995)	0.111	2.139 (1.227-3.727)	0.007	1.911 (1.014-3.602)	0.045	2.491 (1.488-4.172)	0.001	1.605 (0.799-3.223)	0.184
Homocysteine (µmol/L)													
	<12.9	1.000 (Reference)		0.889 (0.638-1.239)	0.486	1.000 (Reference)		0.971 (0.716-1.317)	0.848	1.000 (Reference)		0.772 (0.563-1.059)	0.109
	≥12.9	1.531 (0.955-2.453)	0.077	0.900 (0.469-1.725)	0.751	1.506 (0.880-2.576)	0.135	1.023 (0.579-1.808)	0.938	1.157 (0.727-1.841)	0.540	1.576 (0.764-3.255)	0.219

CAD, coronary artery disease; AOR, adjusted odds ratio; 95% CI, 95% confidence interval, HTN, hypertension; DM, diabetes mellitus; BMI body mass index; LDL, low density lipoprotein; MetS, metabolic syndrome.
The adjusted odds ratio on the basis of risk factors, such as age, gender, hypertension, and diabetes mellitus, smoking, hyperlipidemia.
†Folate 4.0 nmol/L and homocysteine 12.9μmol/L are lower than the 15% cut-off for each level in CAD patients and control subjects.

Table S7. Continued

Variables		*VEGF* +1451 CC		*VEGF* +1451 CT+TT		*VEGF* +1612 GG		*VEGF* +1612 GA+AA		*VEGF* 1725 GG		*VEGF* 1725 GA+AA	
		AOR (95% CI)	*P*	AOR (95% CI)	*P*	AOR (95% CI)	*P*	AOR (95% CI)	*P*	AOR (95% CI)	*P*	AOR (95% CI)	*P*
HTN													
	No	1.000 (Reference)		0.923 (0.612-1.392)	0.702	1.000 (Reference)		1.312 (0.869-1.980)	0.196	1.000 (Reference)		1.480 (0.832-2.632)	0.182
	Yes	1.996 (1.412-2.820)	0.0001	1.617 (1.052-2.485)	0.028	2.267 (1.602-3.206)	<0.0001	1.756 (1.136-2.715)	0.011	2.034 (1.499-2.759)	<0.0001	1.911 (1.017-3.591)	0.044
DM													
	No	1.000 (Reference)		0.950 (0.684-1.320)	0.761	1.000 (Reference)		0.848 (0.606-1.186)	0.336	1.000 (Reference)		0.991 (0.615-1.600)	0.972
	Yes	2.679 (1.683-4.265)	<0.0001	1.846 (1.063-3.205)	0.030	1.698 (1.102-2.614)	0.016	4.434 (2.203-8.925)	<0.0001	2.054 (1.397-3.019)	0.0002	8.068 (2.346-27.744)	0.001
Hemoglobin A1c (%)													
	<6.5	1.000 (Reference)		0.871 (0.628-1.206)	0.405	1.000 (Reference)		0.920 (0.659-1.284)	0.624	1.000 (Reference)		1.061 (0.653-1.724)	0.811
	≥6.5	4.044 (2.186-7.481)	<0.0001	3.453 (1.607-7.423)	0.002	4.479 (2.483-8.080)	<0.0001	7.182 (3.116-16.556)	<0.0001	4.479 (2.660-7.543)	<0.0001	7.099 (2.269-22.212)	0.001
Smoking status													
	No	1.000 (Reference)		0.750 (0.522-1.078)	0.121	1.000 (Reference)		0.995 (0.686-1.444)	0.981	1.000 (Reference)		1.291 (0.749-2.223)	0.358
	Yes	0.907 (0.602-1.367)	0.640	1.217 (0.704-2.102)	0.482	0.860 (0.567-1.306)	0.480	0.962 (0.571-1.620)	0.883	0.917 (0.636-1.321)	0.641	1.189 (0.580-2.436)	0.637
BMI													
	<25	1.000 (Reference)		1.254 (0.872-1.804)	0.222	1.000 (Reference)		0.943 (0.645-1.377)	0.760	1.000 (Reference)		1.210 (0.704-2.080)	0.489
	≥25	3.530 (2.431-5.127)	<0.0001	2.136 (1.322-3.453)	0.002	2.579 (1.804-3.687)	<0.0001	3.135 (1.915-5.133)	<0.0001	2.797 (2.029-3.855)	<0.0001	3.340 (1.650-6.761)	0.001
Hyperlipidemia													
	No	1.000 (Reference)		0.789 (0.561-1.111)	0.175	1.000 (Reference)		0.999 (0.701-1.423)	0.994	1.000 (Reference)		1.150 (0.698-1.895)	0.584
	Yes	1.147 (0.781-1.683)	0.485	1.436 (0.833-2.477)	0.193	1.312 (0.888-1.938)	0.173	1.513 (0.912-2.509)	0.109	1.312 (0.935-1.841)	0.116	1.990 (0.877-4.513)	0.100
LDL-cholesterol (mg/dL)													
	<130	1.000 (Reference)		0.846 (0.600-1.191)	0.337	1.000 (Reference)		1.065 (0.748-1.516)	0.727	1.000 (Reference)		1.231 (0.747-2.029)	0.414
	≥130	3.916 (2.269-6.758)	<0.0001	7.427 (3.299-16.718)	<0.0001	3.637 (2.145-6.169)	<0.0001	8.278 (3.640-18.827)	<0.0001	3.858 (2.402-6.196)	<0.0001	10.962 (3.633-33.078)	<0.0001
MetS													
	No	1.000 (Reference)		1.084 (0.709-1.658)	0.709	1.000 (Reference)		0.863 (0.557-1.330)	0.510	1.000 (Reference)		1.118 (0.595-2.102)	0.729
	Yes	2.781 (1.921-4.026)	<0.0001	2.327 (1.443-3.751)	0.001	2.216 (1.541-3.188)	<0.0001	2.539 (1.566-4.119)	0.0002	2.482 (1.791-3.439)	<0.0001	2.538 (1.315-4.896)	0.006
Folate (nmol/L)													
	≥4.0	1.000 (Reference)		0.927 (0.674-1.275)	0.642	1.000 (Reference)		0.950 (0.686-1.316)	0.758	1.000 (Reference)		1.209 (0.763-1.916)	0.418
	<4.0	2.492 (1.506-4.125)	0.0004	1.758 (0.842-3.670)	0.133	1.680 (1.026-2.750)	0.039	3.062 (1.423-6.590)	0.004	2.010 (1.294-3.122)	0.002	3.204 (0.960-10.698)	0.058
Homocysteine (μmol/L)													
	<12.9	1.000 (Reference)		0.850 (0.618-1.169)	0.317	1.000 (Reference)		1.071 (0.770-1.489)	0.683	1.000 (Reference)		1.170 (0.729-1.876)	0.515
	≥12.9	1.252 (0.789-1.988)	0.340	1.486 (0.715-3.086)	0.289	1.302 (0.811-2.088)	0.274	1.167 (0.621-2.193)	0.632	1.198 (0.786-1.826)	0.401	1.809 (0.705-4.642)	0.218

CAD, coronary artery disease; AOR, adjusted odds ratio; 95% CI, 95% confidence interval, HTN, hypertension; DM, diabetes mellitus; BMI body mass index; LDL, low density lipoprotein; MetS, metabolic syndrome.
The adjusted odds ratio on the basis of risk factors, such as age, gender, hypertension, and diabetes mellitus, smoking, hyperlipidemia.
†Folate 4.0 nmol/L and homocysteine 12.9μmol/L are lower than the 15% cut-off for each level in CAD patients and control subjects.

Table S8. Clinical variables in CAD patients and control subjects stratified by *VEGF* polymorphisms status by ANOVA

Genotypes	BMI (kg/m2)	Total Cholesterol (mg/dL)	Triglyceride (mg/dL)	HDL-Cholesterol (mg/dL)	LDL-Cholesterol (mg/dL)	FBS (mg/dL)	Hemoglobin A1c (%)	SBP (mmHg)	DBP (mmHg)	Folate (mg/mL)	Vitamin B12 (pg/mL)	Homocysteine (mmol/L)	Creatinine (mg/dL)
	Mean ± SD	Mean ± SD	Mean ± SD	Mean ± SD	Mean ± SD	Mean ± SD	Mean ± SD	Mean ± SD	Mean ± SD	Mean ± SD	Mean ± SD	Mean ± SD	Mean ± SD
VEGF -1154G>A													
GG	24.74 ± 3.42	189.91 ± 42.42	154.75 ± 105.82	44.81 ± 12.58	115.01 ± 41.90	130.11 ± 54.50	6.59 ± 2.79	129.57 ± 19.78	79.14 ± 12.30	8.57 ± 7.55	675.99 ± 282.74	9.82 ± 4.88	1.34 ± 5.71
GA	23.90 ± 3.42	181.47 ± 37.33	131.82 ± 61.12	43.30 ± 10.91	114.31 ± 30.62	114.59 ± 53.70	6.44 ± 2.11	133.00 ± 21.54	83.00 ± 9.95	7.14 ± 3.94	498.56 ± 122.62	10.28 ± 4.32	0.94 ± 0.26
AA	24.85 ± 3.20	188.16 ± 43.84	148.82 ± 88.26	44.63 ± 10.61	112.09 ± 37.55	124.75 ± 52.62	6.36 ± 1.44	129.97 ± 19.28	80.49 ± 12.66	8.89 ± 11.03	682.81 ± 274.69	9.91 ± 4.59	1.05 ± 0.64
P	0.587	0.651	0.515	0.898	0.737	0.252	0.671	0.752	0.179	0.678	0.157	0.906	0.719
VEGF -1498T>C													
TT	24.72 ± 3.56	189.86 ± 43.44	151.85 ± 90.55	44.62 ± 12.40	113.29 ± 40.65	128.46 ± 54.46	6.61 ± 3.05	129.77 ± 19.71	79.33 ± 12.54	8.60 ± 7.91	673.08 ± 281.51	9.85 ± 4.51	1.43 ± 6.36
TC	24.64 ± 2.81	188.30 ± 40.31	133.63 ± 88.51	43.13 ± 10.13	127.61 ± 38.89	125.12 ± 65.28	6.43 ± 1.75	134.67 ± 18.88	82.12 ± 12.43	9.59 ± 7.23	719.81 ± 274.48	8.95 ± 3.22	0.94 ± 0.33
CC	24.81 ± 3.11	188.50 ± 41.91	156.62 ± 116.05	45.09 ± 11.76	114.12 ± 40.59	128.73 ± 51.86	6.42 ± 1.51	129.08 ± 19.67	79.62 ± 12.08	8.52 ± 9.57	670.63 ± 277.11	9.98 ± 5.35	1.03 ± 0.55
P	0.929	0.894	0.356	0.690	0.198	0.920	0.700	0.215	0.364	0.758	0.687	0.422	0.459
VEGF +936C>T													
CC	24.87 ± 3.53	187.85 ± 43.80	151.16 ± 103.88	44.98 ± 12.30	113.60 ± 40.02	125.25 ± 50.43	6.36 ± 1.42	129.58 ± 19.92	79.54 ± 12.15	8.49 ± 7.47	660.77 ± 265.66	9.79 ± 4.69	1.38 ± 5.94
CT	24.55 ± 2.99	191.44 ± 40.62	155.67 ± 95.67	43.70 ± 11.24	115.50 ± 42.10	134.45 ± 59.93	6.96 ± 4.08	129.55 ± 19.14	79.42 ± 12.79	8.92 ± 10.87	719.12 ± 309.76	10.05 ± 5.16	1.03 ± 0.65
TT	24.26 ± 3.10	197.48 ± 38.03	156.97 ± 84.13	49.64 ± 13.57	116.42 ± 40.23	134.52 ± 61.18	6.02 ± 0.87	134.68 ± 19.41	81.61 ± 12.93	8.63 ± 4.61	548.83 ± 168.40	9.42 ± 3.18	0.96 ± 0.32
P	0.405	0.293	0.812	0.094	0.845	0.163	0.349	0.365	0.641	0.800	0.017	0.673	0.584
VEGF +1451C>T													
CC	24.86 ± 3.51	188.33 ± 43.51	152.28 ± 104.49	44.90 ± 12.19	114.14 ± 39.74	126.03 ± 51.58	6.38 ± 1.50	129.39 ± 19.79	79.56 ± 12.10	8.38 ± 7.37	670.43 ± 276.83	9.80 ± 4.78	1.37 ± 5.87
CT	24.56 ± 3.04	190.38 ± 41.49	154.17 ± 93.88	43.62 ± 11.38	114.18 ± 42.99	133.53 ± 58.56	6.94 ± 4.13	129.82 ± 19.49	79.29 ± 12.99	9.22 ± 11.24	704.30 ± 293.30	10.02 ± 5.01	1.03 ± 0.67
TT	24.19 ± 2.89	197.76 ± 36.16	150.12 ± 83.30	51.22 ± 13.68	117.32 ± 40.01	131.50 ± 59.30	6.04 ± 0.90	135.56 ± 18.53	81.85 ± 12.40	8.59 ± 4.46	547.67 ± 169.08	9.53 ± 3.29	0.97 ± 0.31
P	0.372	0.408	0.959	0.029	0.946	0.179	0.215	0.205	0.525	0.451	0.050	0.776	0.625
VEGF +1612G>A													
GG	24.70 ± 3.27	188.98 ± 42.15	154.37 ± 107.29	44.67 ± 12.37	114.19 ± 40.89	128.16 ± 52.69	6.50 ± 2.78	129.83 ± 18.97	79.45 ± 12.06	8.88 ± 9.52	667.27 ± 287.34	9.86 ± 4.93	1.35 ± 5.77
GA	25.03 ± 3.53	192.78 ± 48.31	157.59 ± 101.60	44.71 ± 8.11	102.68 ± 40.02	118.11 ± 39.43	6.53 ± 1.29	128.21 ± 21.14	79.82 ± 14.04	7.68 ± 5.23	614.57 ± 266.13	10.01 ± 3.78	0.99 ± 0.25
AA	24.84 ± 3.57	189.66 ± 43.53	147.93 ± 81.64	44.92 ± 11.71	115.71 ± 39.89	130.19 ± 58.68	6.59 ± 1.74	129.71 ± 21.30	79.86 ± 12.97	8.06 ± 5.71	700.74 ± 257.29	9.82 ± 4.54	1.05 ± 0.70
P	0.816	0.891	0.685	0.973	0.416	0.537	0.938	0.913	0.903	0.398	0.358	0.977	0.685
VEGF +1725G>A													
GG	24.72 ± 3.31	189.46 ± 42.92	152.77 ± 102.96	44.79 ± 12.07	114.48 ± 41.04	127.63 ± 52.84	6.48 ± 2.56	129.78 ± 19.29	79.58 ± 12.37	8.70 ± 8.89	674.07 ± 281.16	9.86 ± 4.88	1.29 ± 5.15
GA	28.33 ± 4.09	182.67 ± 36.67	152.67 ± 2.89	39.67 ± 1.44	84.00 ± 79.88	101.00 ± 15.59	7.37 ± 0.29	136.67 ± 5.77	80.00 ± 0.00	3.61 ± 1.53	646.00	17.53 ± 1.67	1.10 ± 0.17
AA	24.87 ± 3.68	188.05 ± 41.35	152.43 ± 83.75	44.54 ± 12.32	113.70 ± 35.11	135.19 ± 62.81	6.85 ± 2.04	129.29 ± 22.65	79.48 ± 12.56	8.16 ± 5.28	679.95 ± 267.98	9.53 ± 3.89	1.02 ± 0.32
P	0.168	0.920	1.000	0.757	0.429	0.290	0.475	0.808	0.995	0.503	0.984	0.017	0.875

CAD, coronary artery disease; BMI body mass index; HDL, high density lipoprotein; LDL, low density lipoprotein; FBS, fasting blood sugar; SBP, systolic blood pressure; DBP, diastolic blood pressure.

Table S9. Clinical variables in CAD patients stratified by *VEGF* polymorphisms status by ANOVA

Genotypes	BMI (kg/m2)	Total Cholesterol (mg/dL)	Triglyceride (mg/dL)	HDL-Cholesterol (mg/dL)	LDL-Cholesterol (mg/dL)	FBS (mg/dL)	Hemoglobin A1c (%)	SBP (mmHg)	DBP (mmHg)	Folate (mg/mL)	Vitamin B12 (pg/mL)	Homocysteine (mmol/L)	Creatinine (mg/dL)
	Mean ± SD	Mean ± SD	Mean ± SD	Mean ± SD	Mean ± SD	Mean ± SD	Mean ± SD	Mean ± SD	Mean ± SD	Mean ± SD	Mean ± SD	Mean ± SD	Mean ± SD
VEGF -1154G>A													
GG	24.99 ± 3.78	186.48 ± 46.10	159.45 ± 114.58	43.90 ± 11.31	112.91 ± 40.22	142.70 ± 61.91	6.53 ± 1.58	128.41 ± 21.52	78.81 ± 12.88	8.47 ± 7.44	659.74 ± 337.24	9.79 ± 5.11	1.69 ± 7.75
GA	25.05 ± 3.00	187.95 ± 49.47	157.23 ± 91.68	43.87 ± 11.07	113.22 ± 40.03	139.61 ± 65.98	6.44 ± 1.40	127.05 ± 20.69	79.29 ± 12.78	9.31 ± 14.71	695.11 ± 349.57	10.33 ± 5.95	1.14 ± 0.87
AA	24.70 ± 3.40	184.50 ± 43.97	146.00 ± 67.51	45.26 ± 10.30	113.90 ± 33.61	123.80 ± 69.68	6.79 ± 2.24	132.00 ± 21.50	82.00 ± 7.89	7.81 ± 4.38	325.00 ± ?	8.99 ± 3.92	0.98 ± 0.30
P	0.956	0.948	0.917	0.930	0.995	0.606	0.780	0.715	0.710	0.722	0.560	0.569	0.555
VEGF -1498T>C													
TT	25.18 ± 3.75	185.82 ± 47.83	155.02 ± 87.39	43.69 ± 11.04	112.01 ± 41.45	142.40 ± 64.61	6.55 ± 1.62	128.13 ± 21.28	78.71 ± 13.02	8.61 ± 8.04	634.51 ± 329.38	9.91 ± 4.63	1.88 ± 8.73
TC	24.79 ± 3.42	188.87 ± 46.21	164.31 ± 134.54	44.48 ± 11.65	113.85 ± 38.43	140.54 ± 58.46	6.47 ± 1.41	127.30 ± 21.26	78.85 ± 12.54	8.73 ± 11.92	713.76 ± 362.26	10.06 ± 6.32	1.08 ± 0.72
CC	24.35 ± 2.69	181.85 ± 38.83	156.95 ± 111.43	42.18 ± 9.78	119.10 ± 32.85	138.68 ± 80.69	6.47 ± 1.83	136.00 ± 21.13	84.00 ± 10.46	8.55 ± 4.24	631.75 ± 256.34	8.37 ± 3.44	0.97 ± 0.29
P	0.393	0.714	0.680	0.600	0.706	0.937	0.903	0.224	0.199	0.992	0.557	0.418	0.436
VEGF +936C>T													
CC	25.18 ± 3.88	185.43 ± 48.17	154.61 ± 110.82	44.04 ± 11.54	113.15 ± 41.83	137.30 ± 59.48	6.47 ± 1.51	128.97 ± 22.07	79.41 ± 12.83	8.07 ± 6.33	609.16 ± 317.66	9.86 ± 5.29	1.74 ± 8.12
CT	24.60 ± 2.87	189.49 ± 43.64	166.20 ± 103.29	43.20 ± 9.94	112.76 ± 35.07	148.41 ± 67.88	6.66 ± 1.68	125.75 ± 19.01	77.72 ± 12.31	10.03 ± 14.94	776.55 ± 363.13	10.09 ± 5.47	1.13 ± 0.88
TT	24.74 ± 3.25	191.07 ± 47.75	173.86 ± 106.06	48.20 ± 14.63	112.14 ± 43.11	165.71 ± 79.26	6.21 ± 0.91	133.57 ± 23.73	82.14 ± 15.28	8.94 ± 5.99	796.00 ± 0.00	9.23 ± 3.93	1.12 ± 0.42
P	0.284	0.662	0.508	0.438	0.992	0.081	0.478	0.211	0.281	0.175	0.080	0.811	0.659
VEGF +1451C>T													
CC	25.19 ± 3.86	185.79 ± 48.13	155.41 ± 112.01	44.10 ± 11.54	113.37 ± 41.67	138.28 ± 60.99	6.50 ± 1.58	129.05 ± 22.10	79.51 ± 12.89	8.04 ± 6.31	617.46 ± 320.29	9.83 ± 5.26	1.73 ± 8.06
CT	24.57 ± 2.87	188.75 ± 43.67	164.65 ± 100.09	43.04 ± 9.90	112.20 ± 35.28	146.48 ± 65.26	6.60 ± 1.53	125.47 ± 18.81	77.43 ± 12.10	10.18 ± 15.10	768.96 ± 366.97	10.16 ± 5.55	1.13 ± 0.89
TT	24.74 ± 3.25	191.07 ± 47.75	173.86 ± 106.06	48.20 ± 14.63	112.14 ± 43.11	165.71 ± 79.26	6.21 ± 0.91	133.57 ± 23.73	82.14 ± 15.28	8.94 ± 5.99	796.00 ± 0.00	9.23 ± 3.93	1.12 ± 0.42
P	0.255	0.784	0.621	0.377	0.960	0.159	0.682	0.168	0.185	0.128	0.136	0.745	0.671
VEGF +1612G>A													
GG	24.84 ± 3.68	184.90 ± 46.99	161.77 ± 117.76	43.46 ± 10.96	112.21 ± 39.91	140.74 ± 60.70	6.44 ± 1.44	127.96 ± 19.83	78.78 ± 12.35	9.13 ± 10.64	676.42 ± 363.99	9.92 ± 5.65	1.73 ± 8.00
GA	25.25 ± 3.40	192.18 ± 47.34	152.41 ± 85.22	45.01 ± 12.15	116.44 ± 39.68	146.25 ± 70.18	6.71 ± 1.82	128.48 ± 24.59	79.31 ± 13.61	7.77 ± 6.82	658.87 ± 291.46	9.79 ± 4.45	1.12 ± 0.94
AA	25.92 ± 3.28	183.00 ± 38.48	145.94 ± 77.57	44.72 ± 8.71	103.38 ± 43.14	123.11 ± 46.04	6.50 ± 1.25	129.44 ± 22.87	80.56 ± 14.34	6.65 ± 5.77	514.40 ± 114.28	10.48 ± 4.35	0.98 ± 0.26
P	0.307	0.321	0.633	0.413	0.384	0.321	0.313	0.941	0.804	0.291	0.590	0.873	0.647
VEGF +1725G>A													
GG	24.94 ± 3.57	186.59 ± 47.28	158.38 ± 110.71	43.70 ± 11.03	113.21 ± 40.04	141.06 ± 61.86	6.44 ± 1.44	128.18 ± 20.54	78.86 ± 12.65	8.81 ± 10.02	678.50 ± 355.25	9.92 ± 5.47	1.61 ± 7.14
GA	25.25 ± 3.68	188.46 ± 44.48	160.54 ± 95.25	45.70 ± 12.60	113.21 ± 37.11	147.05 ± 71.53	7.01 ± 2.14	127.54 ± 26.54	79.89 ± 13.91	7.87 ± 6.04	580.29 ± 230.40	9.38 ± 3.62	1.04 ± 0.39
AA	28.23 ± 4.22	182.67 ± 36.67	152.67 ± 2.89	39.67 ± 1.44	84.00 ± 79.88	101.00 ± 15.59	7.37 ± 0.29	136.67 ± 5.77	80.00 ± 0.00	3.61 ± 1.53	646.00	17.53 ± 1.67	1.10 ± 0.17
P	0.243	0.950	0.986	0.367	0.452	0.430	0.057	0.258	0.840	0.532	0.813	0.033	0.831

CAD, coronary artery disease; BMI body mass index; HDL, high density lipoprotein; LDL, low density lipoprotein; FBS, fasting blood sugar; SBP, systolic blood pressure; DBP, diastolic blood pressure.

Table S10. Clinical variables in control subjects stratified by *VEGF* polymorphisms status by ANOVA

Genotypes	BMI (kg/m2)	Total Cholesterol (mg/dL)	Triglyceride (mg/dL)	HDL-Cholesterol (mg/dL)	LDL-Cholesterol (mg/dL)	FBS (mg/dL)	Hemoglobin A1c (%)	SBP (mmHg)	DBP (mmHg)	Folate (mg/mL)	Vitamin B12 (pg/mL)	Homocysteine (mmol/L)	Creatinine (mg/dL)
	Mean ± SD	Mean ± SD	Mean ± SD	Mean ± SD	Mean ± SD	Mean ± SD	Mean ± SD	Mean ± SD	Mean ± SD	Mean ± SD	Mean ± SD	Mean ± SD	Mean ± SD
VEGF -1154G>A													
GG	24.15 ± 3.26	193.94 ± 37.30	149.21 ± 94.35	47.16 ± 15.19	120.37 ± 45.64	115.54 ± 39.80	6.76 ± 4.91	130.92 ± 17.49	79.52 ± 11.60	8.67 ± 7.68	680.22 ± 267.30	9.85 ± 4.61	0.93 ± 0.22
GA	24.56 ± 3.47	188.36 ± 37.95	140.83 ± 84.50	46.26 ± 9.46	109.73 ± 32.03	111.50 ± 31.74	6.21 ± 1.52	132.66 ± 17.54	81.60 ± 12.49	8.54 ± 6.68	680.85 ± 262.55	9.53 ± 2.88	0.98 ± 0.27
AA	21.93 ± 2.98	177.14 ± 27.91	111.57 ± 48.07	36.77 ± 12.40	115.67 ± 23.18	101.43 ± 8.00	5.05 ± 0.07	134.25 ± 23.00	84.25 ± 12.54	6.30 ± 3.42	520.25 ± 111.10	11.88 ± 4.51	0.89 ± 0.20
P	0.247	0.225	0.421	0.418	0.308	0.406	0.676	0.597	0.169	0.665	0.237	0.287	0.22
VEGF -1498T>C													
TT	24.99 ± 3.01	193.91 ± 41.57	113.35 ± 57.52	45.49 ± 11.29	148.88 ± 46.69	113.91 ± 48.21	6.27 ± 1.67	133.52 ± 17.08	80.48 ± 13.93	10.35 ± 8.85	735.13 ± 280.04	9.42 ± 3.02	0.92 ± 0.36
TC	23.95 ± 3.21	194.41 ± 37.51	148.29 ± 94.03	46.93 ± 15.09	116.52 ± 38.58	113.11 ± 34.57	6.80 ± 5.50	131.58 ± 17.69	80.01 ± 11.99	8.59 ± 7.79	682.22 ± 268.92	9.78 ± 4.38	0.93 ± 0.22
CC	24.58 ± 3.53	188.09 ± 36.52	147.81 ± 89.99	46.59 ± 11.99	114.75 ± 45.44	115.76 ± 39.79	6.30 ± 1.72	131.05 ± 17.59	80.47 ± 11.53	8.32 ± 6.36	661.05 ± 254.88	9.89 ± 4.06	0.97 ± 0.25
P	0.224	0.261	0.206	0.954	0.09	0.792	0.774	0.815	0.924	0.463	0.416	0.877	0.199
VEGF +936C>T													
CC	24.16 ± 3.42	190.67 ± 38.00	147.15 ± 95.25	47.33 ± 13.82	114.73 ± 35.18	111.79 ± 33.13	6.06 ± 1.09	130.26 ± 17.16	79.68 ± 11.34	8.92 ± 8.51	672.99 ± 250.93	9.71 ± 3.94	0.97 ± 0.25
CT	24.47 ± 3.18	193.50 ± 37.20	144.48 ± 85.83	44.86 ± 13.84	121.59 ± 54.50	119.73 ± 46.07	7.71 ± 7.10	133.48 ± 18.54	81.17 ± 13.10	7.91 ± 4.56	705.80 ± 296.09	10.01 ± 4.85	0.92 ± 0.21
TT	23.51 ± 2.88	202.76 ± 28.17	143.06 ± 60.63	53.66 ± 10.27	128.40 ± 31.60	108.82 ± 18.97	5.50 ± 0.55	135.59 ± 15.70	81.18 ± 11.11	8.40 ± 3.39	534.29 ± 161.51	9.58 ± 2.45	0.82 ± 0.10
P	0.615	0.379	0.954	0.28	0.501	0.475	0.119	0.139	0.469	0.435	0.035	0.787	0.004
VEGF +1451C>T													
CC	24.16 ± 3.39	191.21 ± 37.47	148.74 ± 95.31	46.79 ± 13.46	115.93 ± 34.85	112.68 ± 34.22	6.10 ± 1.23	129.76 ± 16.88	79.62 ± 11.18	8.73 ± 8.33	682.91 ± 264.67	9.78 ± 4.20	0.96 ± 0.25
CT	24.53 ± 3.31	192.25 ± 38.96	142.12 ± 85.05	45.21 ± 14.71	119.36 ± 58.74	118.78 ± 45.85	7.98 ± 7.81	134.67 ± 19.16	81.36 ± 13.67	8.27 ± 4.97	688.71 ± 272.29	9.87 ± 4.39	0.92 ± 0.22
TT	23.59 ± 2.43	202.45 ± 25.59	133.50 ± 60.40	59.68 ± 5.04	131.80 ± 28.39	107.55 ± 18.52	5.40 ± 0.62	136.95 ± 14.35	81.65 ± 10.35	8.36 ± 3.28	535.25 ± 163.36	9.75 ± 2.80	0.87 ± 0.13
P	0.553	0.431	0.661	0.082	0.658	0.244	0.03	0.014	0.257	0.842	0.047	0.979	0.119
VEGF +1612G>A													
GG	24.32 ± 3.13	193.41 ± 35.72	146.31 ± 94.12	47.52 ± 14.85	118.91 ± 42.92	114.99 ± 38.66	6.67 ± 4.76	131.84 ± 17.81	80.17 ± 11.72	8.64 ± 8.30	665.21 ± 267.84	9.80 ± 4.06	0.94 ± 0.24
GA	24.21 ± 3.75	186.85 ± 38.86	142.93 ± 77.52	44.69 ± 10.62	113.96 ± 40.75	112.39 ± 34.97	6.24 ± 1.47	131.05 ± 16.98	80.46 ± 12.25	8.36 ± 4.30	709.50 ± 250.15	9.85 ± 4.67	0.96 ± 0.23
AA	21.74 ± 2.37	212.33 ± 61.59	180.89 ± 140.86	44.70 ± 3.45	99.00 ± 20.78	108.11 ± 19.53	6.70 ± 2.12	126.00 ± 18.53	78.50 ± 14.15	9.74 ± 3.34	670.22 ± 314.33	9.17 ± 2.47	1.01 ± 0.24
P	0.23	0.116	0.486	0.547	0.589	0.731	0.87	0.561	0.878	0.843	0.326	0.888	0.52
VEGF +1725G>A													
GG	24.24 ± 3.29	192.56 ± 37.46	146.71 ± 93.65	47.36 ± 13.93	117.48 ± 43.28	113.54 ± 36.41	6.58 ± 4.32	131.48 ± 17.74	80.36 ± 12.04	8.60 ± 7.63	673.13 ± 263.23	9.81 ± 4.19	0.94 ± 0.24
GA	24.20 ± 3.64	187.49 ± 37.07	141.15 ± 63.92	40.67 ± 10.77	115.17 ± 29.31	119.38 ± 44.97	6.36 ± 1.66	131.51 ± 16.49	78.96 ± 10.76	8.50 ± 4.26	712.40 ± 273.74	9.72 ± 4.24	0.99 ± 0.18
AA	0.00 ± 0.00	0.00 ± 0.00	0.00 ± 0.00	0.00 ± 0.00	0.00 ± 0.00	0.00 ± 0.00	0.00 ± 0.00	0.00 ± 0.00	0.00 ± 0.00	0.00 ± 0.00	0.00 ± 0.00	0.00 ± 0.00	0.00 ± 0.00
P	0.94	0.411	0.711	0.056	0.825	0.947	0.846	0.992	0.456	0.933	0.357	0.898	0.173

BMI body mass index; HDL, high density lipoprotein; LDL, low density lipoprotein; FBS, fasting blood sugar; SBP, systolic blood pressure; DBP, diastolic blood pressure.
